# Overexpression of YAP 1 contributes to progressive features and poor prognosis of human urothelial carcinoma of the bladder

**DOI:** 10.1186/1471-2407-13-349

**Published:** 2013-07-19

**Authors:** Jian-Ye Liu, Yong-Hong Li, Huan-Xin Lin, Yi-Ji Liao, Shi-Juan Mai, Zhou-Wei Liu, Zhi-Ling Zhang, Li-Juan Jiang, Jia-Xing Zhang, Hsiang-Fu Kung, Yi-Xin Zeng, Fang-Jian Zhou, Dan Xie

**Affiliations:** 1 State Key Laboratory of Oncology in South China, Cancer Center, Sun Yat-Sen University, No 651, Dongfeng Road East, Guangzhou 510060, China; 2 Department of Urology, Cancer Center, Sun Yat-Sen University, No 651, Dongfeng Road East, Guangzhou 510060, China; 3 Department of Pathology, Cancer Center, Sun Yat-Sen University, No 651, Dongfeng Road East, Guangzhou 510060, China

**Keywords:** Urothelial carcinoma of the bladder, YAP 1, Immunohistochemistry, Prognosis

## Abstract

**Background:**

Yes-associated protein 1 (YAP 1), the nuclear effector of the Hippo pathway, is a key regulator of organ size and a candidate human oncogene in multiple tumors. However, the expression dynamics of YAP 1 in urothelial carcinoma of the bladder (UCB) and its clinical/prognostic significance are unclear.

**Methods:**

In this study, the methods of quantitative real-time polymerase chain reaction (qRT-PCR), Western blotting and immunohistochemistry (IHC) were utilized to investigate mRNA/ protein expression of YAP 1 in UCBs. Spearman’s rank correlation, Kaplan-Meier plots and Cox proportional hazards regression model were used to analyze the data.

**Results:**

Up-regulated expression of YAP 1 mRNA and protein was observed in the majority of UCBs by qRT-PCR and Western blotting, when compared with their paired normal bladder tissues. By IHC, positive expression of YAP 1 was examined in 113/213 (53.1%) of UCBs and in 6/86 (7.0%) of normal bladder specimens tissues. Positive expression of YAP 1 was correlated with poorer differentiation, higher T classification and higher N classification (*P* < 0.05). In univariate survival analysis, a significant association between positive expression of YAP 1 and shortened patients’ survival was found (*P* < 0.001). In different subsets of UCB patients, YAP 1 expression was also a prognostic indicator in patients with grade 2 (*P* = 0.005) or grade 3 (*P* = 0.046) UCB, and in patients in pT1 (*P* = 0.013), pT2-4 (*P* = 0.002), pN- (*P* < 0.001) or pT2-4/pN- (*P* = 0.004) stage. Importantly, YAP 1 expression (*P* = 0.003) together with pT and pN status (*P*< 0.05) provided significant independent prognostic parameters in multivariate analysis.

**Conclusions:**

Our findings provide evidences that positive expression of YAP 1 in UCB may be important in the acquisition of an aggressive phenotype, and it is an independent biomarker for poor prognosis of patients with UCB.

## Background

Bladder cancer is one of the most lethal urological malignant tumors worldwide [1]. Urothelial carcinoma of the bladder (UCB) is the most common histological subtype of bladder cancer. Overall, 70% of bladder tumors present as noninvasive urothelial carcinoma (UC), and the remainder present as muscle-invasive disease [2]. To date, the best established and routinely used clinical markers to predict UCBs prognosis are pTNM stage and tumor differentiation [3]. However, the prognosis of UCB patients with disease of the same clinical stage often differs substantially even after surgical resection, and this large variation is mostly unexplained. Thus, a large amount of investigations on UCB have focused on the discovery of specific molecular markers that could serve as reliable prognostic factors. To date, however, the search for specific molecules in UCB cells that have clinical/prognostic value remains substantially limited.

Yes-associated protein 1 (YAP 1), a 65-kDa proline-rich phosphorprotein, is one of the transcription co-activator which is regulated by the Hippo tumor suppressor pathway [4-8]. YAP 1 was originally identified because of its interaction with the Src family tyrosine kinase Yes [9,10]. Recently, YAP 1 has been suggested to be a candidate oncogene [11-13], and it was found to be elevated in several types of cancers including liver, colon, prostate, ovarian, and breast cancers [14-16]. In addition, it was reported that transgenic mice with liver-specific YAP 1 overexpression showed a dramatic increase in liver size and eventually developed tumors [17,18]. To date, however, abnormalities in YAP 1 and their clinicopathologic/prognostic implication in UCBs have not been explored.

In this study, quantitative real-time polymerase chain reaction (qRT-PCR), western blotting, immunohistochemistry (IHC) and tissue microarray (TMA) were utilized to examine the expression dynamics of YAP 1 in a cohort of UCB and normal bladder tissues. In addition, the correlation between expression of YAP 1 and cell proliferation levels in UCB tissue was analyzed using the Ki-67 assessment marker.

## Methods

### Patients and primary UCB samples

For qRT-PCR and western blot analysis, we collected 14 paired fresh UCBs and normal tissue samples from patients who underwent surgery between October 2011 and April 2012. In addition, a cohort of 213 formalin-fixed, paraffin–embedded tissues of UCBs diagnosed between 2002 and 2007 at the Department of Pathology and Urology, Cancer Center and the First Affiliated Hospital, Sun Yat-sen University (Guangzhou, China) was retrieved. The cases selected were based on distinctive pathologic diagnosis of UCB, undergoing curative resection for tumor without preoperative chemotherapy and radiotherapy, and availability of resection tissue and follow-up data. The disease stage of each patient was classified or reclassified according to the 2002 AJCC staging system [19]. The 213 patients included 183 males and 30 females aged from 20 to 89 years (median, 62 years). The average follow-up time was 86.36 months (range, 56.0 to 120.0 months). Among these patients, 89 underwent radical cystectomy (RC) and 124 underwent transurethral resection of bladder tumor (TURBT). After TURBT, 50 mg THP was used in intravesical therapy as weekly intravesical injection beginning within 24 hours after surgery. The clinicopathological characteristics of these 213 patients are summarized in Table 1. The patients’ consent was obtained for the use of the tissue samples and records, and the study protocol was approved and permission for use of the clinical data was given by the Institutional Research Ethics Committee of Sun Yat-Sen University Cancer Center.

**Table 1:** Correlation between YAP 1 expression and clinicopathological characteristics of UCB patients

**Characteristics**	**YAP 1 protein**			***P* value**^**a**^
	**Total cases**	**Negative no (%)**	**Positive no (%)**	
Age (years)				0.604
≦62^b^	111	54(48.6)	57(51.4)	
>62	102	46(45.1)	56(54.9)	
Gender				0.450
Male	183	84(45.9)	99(54.1)	
Female	30	16(53.3)	14(46.7)	
Histological grade				**0.001**
G1	77	49(63.6)	28(36.4)	
G2	69	29(42.0)	40(58.0)	
G3	67	22(32.8)	45(67.2)	
pT classification				**0.010**
pTa/pTis	89	52(58.4)	37(41.6)	
pT1	42	19(45.2)	23(54.8)	
pT2-4	82	29(35.4)	53(64.6)	
pN classification				**0.028**
pN-	195	96(49.2)	99(50.8)	
pN+	18	4(22.2)	14(77.8)	
Tumor size (cm)				0.113
≦2.4^c^	107	56(52.3)	51(47.7)	
>2.4	106	44(41.5)	62(58.5)	
Tumor multiplicity				0.561
Unifocal	102	50(49.0)	52(51.0)	
Multifocal	111	50(45.0)	61(55.0)	

^a^Chi-square test. ^b^median age. ^c^mean size. UCB: urothelial carcinoma of the bladder.

### qRT-PCR analysis

Total RNA was isolated from the 14 pairs of UCB tissue and normal bladder tissue using TRIZOL reagent (Invitrogen, Carlsbad, CA). RNA was reverse-transcribed using SuperScript First Strand cDNA System (Invitrogen, Carlsbad, CA) according to the manufacturer’s instructions. The YAP 1 sense primer was 5′-CGCTCTTCAACGCCGTCA-3′, and the antisense primer was 5′-AGTACTGGCCTGTCGGGAGT-3′. For the β-actin gene, the sense primer was 5′-ATAGCACAGCCTGGATAGCAACGTAC-3′, and the antisense primer was 5′-CACCTTCTACAATGAGCTGCGTGTG-3′. qRT-PCR was done using SYBR Green PCR master mix (Applied Biosystems) in a total volume of 20 μl on the 7900HT fast Real-time PCR system (Applied Biosystems) as follows: 50°C for 2 min, 95°C for 10 min, 40 cycles of 95°Cfor 15 s, and 60°C for 60 s. A dissociation procedure was performed to generate a melting curve for confirmation of amplification specificity. β-actin was used as the reference gene. The relative levels of gene expression were represented asΔCt =Ct_gene_- Ct_reference,_ and the fold change of gene expression was calculated by the 2^-ΔΔCt^ Method. Experiments were repeated in triplicate.

### Western blot analysis

Total proteins from the 14 pairs of UCB tissues and normal bladder tissues were extracted with 1× SDS sample buffer [62.5 mmol/L Tris–HCl (pH 6.8), 2% SDS, 10% glycerol, and 5% 2-mercaptoethanol], and 30 μg of each protein was electrophoretically separated on 12% SDS polyacrylamide gels, and transferred to polyvinylidene difluoride membranes (Millipore). Mouse monoclonal anti-YAP 1(1:300, Upstate Biotechnology, Lake Placid, NY) and anti-mouse (1:2000, Santa Cruz Biotechnology, Santa Cruz, CA) antibodies were used to detect the YAP 1 protein. Mouse GAPDH (1:2000, Sigma) and anti-mouse (1:2000, Santa Cruz Biotechnology, Santa Cruz, CA) antibodies were used to detect GAPDH.

### TMA construction

TMA was constructed as the method described previously [20]. In brief, formalin-fixed, paraffin-embedded tissue blocks and the corresponding hematoxylin and eosin (H&E)-stained slides were over laid for TMA sampling. The slides were reviewed by a pathologist to determine and mark out representative tumor areas. Duplicate of 0.6 mm diameter cylinders were punched from representative tumor areas of individual donor tissue block, and re-embedded into a recipient paraffin block at a defined position, using a tissue arraying instrument (Beecher Instruments, SilverSpring, MD, USA). In our constructed bladder tissue-TMA, three cores of a sample were selected from each primary UCB and normal bladder tissue. Multiple sections (5 μm thick) were cut from the TMA block and mounted on microscope slides. The TMA block contained 213 UCBs and 86 specimens of normal bladder tissues.

### Immunohistochemistry (IHC)

The TMA slides were dried overnight at 37°C, deparaffinized in xylene, rehydrated through graded alcohol, immersed in 3% hydrogen peroxide for 15 minutes to block endogenous peroxidase activity. And antigen-retrieved by pressure cooking for 4 minutes in 10 nmol/l citrate buffer (pH = 6.0) for YAP 1, or in ethylene-diamine tetraacetic acid (EDTA) buffer (pH = 8.0) for Ki-67. Then the slides were preincubated with 10% normal goat serum at room temperature for 30 minutes to reduce nonspecific reaction. Subsequently, the slides were incubated with mouse monoclonal anti-YAP 1 (Upstate Biotechnology, Lake Placid, NY) at a concentration of 3 μg/ml and mouse monoclonal anti-Ki-67 (1:100, Zymed Laboratories Inc., South San Francisco, CA) overnight at 4°C. The slides were sequentially incubated with a secondary antibody (Envision; Dako, Glostrup, Denmark) for 2 hours and 30 minutes at room temperature, and stained with DAB (3,3-diaminobenzidine). Finally, the sections were counterstained with Mayer’s hematoxylin, dehydrated, and mounted. A negative control was obtained by replacing the primary antibody with a normal murine IgG. Known immunostaining positive slides were used as positive controls.

### IHC evaluation

Two independent, blinded investigators examined all tumor slides randomly. Five views were examined per slide, and 100 cells were observed per view at ×400 magnification. We graded the YAP 1 expression according to the distribution, intensity, and percentage of positive cells as described previously [14,21]. Absence of reactivity was graded as negative. With regard to cytoplasmic distribution, weak cytoplasmic reactivity was considered as low expression regardless of extent. Strong cytoplasmic reactivity with less than 50% positive cells was graded as low expression. Otherwise it was graded as high expression. With regard to nuclear distribution, nuclear expression in less than 10% of cells was graded as low expression and nuclear expression in more than 10% cells was graded as high expression. Samples with low or high YAP 1 staining were classified as YAP 1 positive expression. The status of nuclear expression of Ki-67 was assessed by determining the percentage of positive cells stained in each tissue section.

### Statistical analysis

Statistical analysis was performed using the SPSS statistical software package (standard version 13.0; SPSS, Chicago, IL). The association of YAP 1 expression with UCB patient’s clinic-pathological features and the molecular feature Ki-67 was assessed using the χ^2^-test. For survival analysis, we analyzed all UCB patients using Kaplan-Meier analysis. Log-rank test was used to compare different survival curves. Univariate and multivariate survival analyses were performed using the Cox proportional hazards regression model. Multivariate survival analysis was performed on all parameters that were found to be significant on univariate analysis. Differences were considered significant if the *P*-value from a two-tailed test was <0.05.

## Results

### Expression of YAP 1 mRNA by qRT-PCR and YAP 1 protein expression by Western blotting in paired bladder tissues

**Figure 1:**
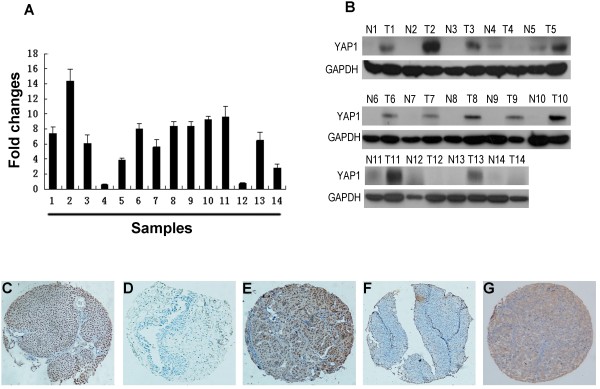
**The expression of YAP 1 in UCB and normal bladder tissues. (A)** Up-regulated expression of YAP 1 mRNA was examined by qRT-PCR in 12/14 UCB cases, when compared with paired normal bladder tissues. Expression levels were normalized for β-actin. Error bars, SD calculated from three parallel experiments. **(B)** Up-regulated expression of YAP 1 protein was detected by Western blotting in 11/14 UCB cases, when compared with paired normal bladder tissues. Expression levels were normalized with GAPDH. **(C**-**F)** The expression of YAP 1 in UCB and normal bladder tissues by IHC (100×). An UCB (case 39) tissue showed high expression of YAP 1, in which more than 90% of tumor cells were positively stained by YAP 1 in the nucleus **(C)**, while its paired normal bladder urothelial mucosal tissue was negatively stained by YAP 1 **(D)**. High expression of YAP 1 was observed in another UCB tissue (case 102), in which about 70% of tumor cells demonstrated a nuclear staining with a lesser cytoplasmic staining of YAP 1 **(E)**. An UCB (case 78) was examined low expression of YAP 1, in which less than 5% of tumor cells showed nuclear staining of YAP 1 **(F)**. An UCB (case 114) tissue showed high expression of YAP 1, in which more than 90% of tumor cells were positively stained by YAP 1 in the cytoplasm **(G)**.

Our qRT-PCR results showed that YAP1 mRNA expression was upregulated in 12 of the 14 UCB samples compared with the paired normal bladder tissues (Figure 1A). Western blotting analyses also demonstrated upregulation of the YAP 1 protein in 11 of the 14 UCB samples compared to their normal counterparts (Figure 1B).

### Expression of YAP 1 in UCBs as determined by IHC

Next, expression and subcellular localization of the YAP 1 protein were determined by IHC in a TMA representative of 213 cases of UCBs and 86 specimens of normal bladder tissues. IHC staining showed that the YAP 1 protein was mainly accumulated in the nucleus with a lesser cytoplasmic presence in bladder tissues (Figure 1C-1G). Based on the criteria described before, positive expression of YAP 1 was found in 53.1% (113 ⁄ 213) of UCBs, and only 7.0% (6 ⁄ 86) of normal bladder tissues.

### Relationship between YAP 1 expression and UCB patients’ clinicopathologic variables

In our UCB cohort, the relationship between the expression of YAP 1 and patient clinical characteristics was shown in Table 1. Positive expression of YAP 1 was found to significantly correlate with poorer differentiation (*P* = 0.001), higher T classification (*P*=0.010) and higher N classification (*P* = 0.028). No significant difference in YAP 1 expression was observed with age, gender, tumor size and multiplicity (*P* > 0.05).

### Relationship between clinicopathologic features, YAP 1 expression, and UCB patients’ survival: univariate survival analysis

**Figure 2:**
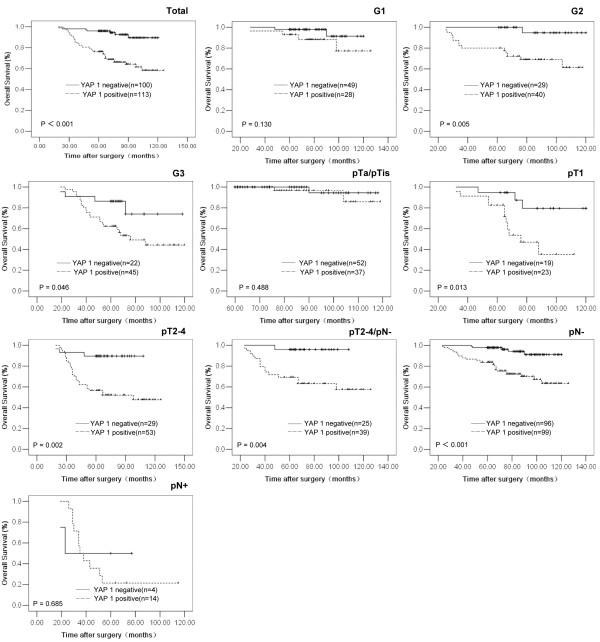
**Kaplan-Meier survival analysis of YAP 1 expression in patients with UCB (log-rank test). ***Total,* probability of survival of all patients with UCB: negative expression (*solid line*), *n* = 100; positive expression (*dashed line*), *n* = 113. *G1,* probability of survival of G1 patients with UCB: negative expression (*solid line*), *n* = 49; positive expression (*dashed line*), *n* = 28. *G2,* probability of survival of G2 patients with UCB: negative expression (*solid line*), *n* = 29; positive expression (*dashed line*), *n* = 40. *G3,* probability of survival of G3 patients with UCB: negative expression (*solid line*), *n* = 22; positive expression (*dashed line*), *n* = 45. *pTa/pTis,* probability of survival of pTa/pTis patients with UCB: negative expression (*solid line*), *n* = 52; positive expression (*dashed line*), *n* = 37. *pT1,* probability of survival of pT1 patients with UCB: negative expression (*solid line*), *n* = 19; positive expression (*dashed line*), *n* = 23. *pT2-4,* probability of survival of pT2-4 patients with UCB: negative expression (*solid line*), *n* = 29; positive expression (*dashed line*), *n* = 53. *pT2-4/pN-,* probability of survival of pT2-4/pN- patients with UCB: negative expression (*solid line*), *n* = 25; positive expression (*dashed line*), *n* = 39. *pN-,* probability of survival of pN- patients with UCB: ngative expression (*solid line*), *n* = 96; positive expression (*dashed line*), *n* = 99. *pN+,* probability of survival of pN+ patients with UCB: negative expression (*solid line*), *n* = 4; positive expression (*dashed line*), *n* = 14.

In univariate survival analyses, cumulative survival curves were calculated according to the Kaplan-Meier method. Differences in survival times were assessed using the log-rank test. First, to confirm the representativeness of the UCBs in our study, we analyzed established prognostic predictors of patient survival. Kaplan-Meier analysis demonstrated a significant impact of well-known clinical pathological prognostic parameters, such as tumor grade, pT status and pN status on patient survival (*P* < 0.05, Table 2). Assessment of survival in total UCBs revealed that positive expression of YAP 1 was correlated with adverse survival of UCB patients (*P* < 0.001, Table 2, Figure 2). Moreover, expression of YAP 1 was found to be a prognostic factor in UCB patients having grades 2 and 3 tumors (*P* = 0.005 and 0.046, respectively, Figure 2, Table 2), pT1 (*P* = 0.013), pT2-4 (*P* = 0.002) and pN- (*P* < 0.001) (Figure 2, Table 2). In addition, survival analysis with regard to YAP 1 expression and a subset of pT2-4 UCB patients without lymph node metastasis (pT2-4/pN-, *n* = 64) showed that expression of YAP1 was also a significant prognostic factor (*P* = 0.004, Figure 2, Table 2).

**Table 2:** Univariate analysis of different prognostic factors in 213 patients with urothelial carcinoma of bladder

**Characteristics**	**Total cases**	**HR (95% CI)**	***P* value**
Age (years)			0.118
≦62^a^	111	1	
>62	102	1.598 (0.888-2.874)	
Gender			0.054
Male	183	1	
Female	30	0.241 (0.058-0.993)	
Histological grade			**<0.001**
G1	77	1	
G2	69	2.627 (1.009-6.840)	
G3	67	6.580 (2.701-16.030)	
pT classification			**<0.001**
pTa/pTis	89	1	
pT1	42	11.433 (3.282-39.828)	
pT2-4	82	14.407 (4.382-47.365)	
pN classification			**<0.001**
pN-	195	1	
pN+	18	9.310 (4.818-17.991)	
Tumor size (cm)			**0.003**
≦2.4^b^	107	1	
>2.4	106	2.572 (1.372-4.823)	
Tumor multiplicity			0.939
Unifocal	102	1	
Multifocal	111	0.978 (0.548-1.744)	
YAP 1			**<0.001**
Negative	100	1	
Positive	113	5.501 (2.460-12.304)	

^a^median age. ^b^mean size. *HR* Hazards ratio. *CI* confidence interval.

### Independent prognostic factors for UCB: multivariate cox regression analysis

Since variables observed to have a prognostic influence by univariate analysis may covariate, the expression of YAP 1 and those clinicalopathological parameters that were significant in univariate analysis (i.e., tumor grade, pT status, pN status, tumor size) were further examined in multivariate analysis. The results showed that the expression of YAP 1 was an independent prognostic factor for overall patient survival (relative risk: 3.553, CI: 1.561-8.086, *P* = 0.003, Table 3). With regard to other parameters, only tumor pT or pN status was shown to be an independent prognostic factor (*P*<0.05, Table 3) for overall survival.

**Table 3:** Multivariate analysis on overall patient survival (Cox regerssion model)

**Characteristic**	**Hazards ratio**	**95% CI**	***P* value**
YAP 1 (Negative vs Positive)	3.553	1.561-8.086	**0.003**
Histological grade (G1 vs G2 vs G3)	1.348	0.861-2.111	0.192
pT classification (pTa/pTis vs pT1 vs pT2-4)	1.668	1.042-2.669	**0.033**
pN classification (pN- vs pN+)	3.827	1.800-8.136	**<0.001**
Tumor size (cm) (≦2.4^a^ vs >2.4)	1.900	0.987-3.657	0.055

^a^mean size. *CI* confidence interval.

### Correlation between expressions of YAP1 and Ki-67

To address whether or not YAP 1 expression in UCB is correlated with cell proliferation, the expression of Ki-67, a widely used cellular proliferation marker, was investigated using IHC in our UCB cohort. The expression level of Ki-67 was assessed as a labeling index (LI), i.e., as the percentage of Ki-67 positive cells in each tumor. In our UCB cohorts, the mean LI value of Ki-67 for all 213 UCB tumor samples was 31.2%, thus, the mean value of 31.2% was used as a cutoff value to define low Ki-67 LI (LI<31.2%) and high Ki-67 LI (LI≧31.2%). A significant positive correlation between expression of YAP 1 and Ki67 was evaluated in our UCB cohort, in which the frequency of cases with high expression of Ki67 was significantly larger in carcinomas with a positive expression of YAP 1 (74/113 cases, 65.9%) than in those cases with a negative expression of YAP 1 (46/100 cases, 46.0%; χ^2^ test, *P* = 0.004, Table 4).

**Table 4:** The correlation between expression of YAP 1 and of Ki-67 in 213 cases of UCB

**YAP 1**	**Cases**	**Labeling index (LI) of Ki-67**		***P* value**^**a**^
		**Low no (%)**	**High no (%)**	
Negative	100	54(54.0)	46(46.0)	**0.004**
Positive	113	39(34.5)	74(65.5)	

^a^Chi-square test. *UCB* urothelial carcinoma of the bladder.

## Discussion

Clinically, pTNM stage and tumor histopathological grade are the best-established predictive factors for important aspects affecting the prognosis of patients with UCB [22]. These two parameters, however, based on specific clinicopathologic features and extent of disease, may have reached their limits in providing critical information influencing patient prognosis and treatment strategies. Furthermore, the outcome of patients with the same stage and/or pathological grade of UCB is substantially different and such large discrepancy has not been explored [23,24]. Thus, there is an urgent need for new objective strategies that can effectively distinguish between patients with favorable and unfavorable prognosis.

YAP 1 is phosphorylated by the Hippo signaling pathway, and is highly conserved along with other components of this pathway; it is involved in regulating the balance between cell proliferation and apoptosis to maintain the steady-state of the cellular environment [5,6,16]. Overexpression of YAP 1 has been implicated in tumor progression in various human cancers, such as liver, colon, ovarian and lung cancers [12,14,15,25]. These findings suggest a potential oncogenic role of YAP1 in multiple human cancers. To date, however, the expression status of YAP 1 in UCBs and its correlation with the clinicopathological factors of this tumor has not been elucidated. In the present study, we first examined the expression of YAP 1, both in mRNA and protein levels, in UCB and paired normal bladder tissues by qRT-PCR and western blotting, respectively. Our results showed that the mRNA and protein expressions of YAP 1 were frequently up-regulated in UCB tissues, when compared with their paired normal bladder tissues. Next, the expression dynamics of the YAP 1 protein was examined by IHC, using a TMA containing a large cohort of UCB and normal bladder tissues. Our results demonstrated that positive expression of YAP 1 was frequently observed in UCB tissues. In contrast, only a small population of normal bladder tissues showed positive staining for YAP 1. These findings suggest the possibility that up-regulated expression of YAP 1 may provide a selective advantage in the UCB tumorigenic processes.

In previous studies, YAP 1 expression was found to be elevated and correlate closely with aggressive features, and/or poor prognosis in many human cancers [14-16,21,26-30]. A clinical study involving 177 hepatocellular carcinoma patients showed that YAP 1 could serve as an independent predictor for hepatocellular carcinoma-specific, disease-free survival and overall survival [15]. In 92 cases of non-small-cell lung carcinoma, positive expression YAP 1 was observed in 66.3% of the cases, and it was significantly correlated with lymph node metastasis and later clinical stages, and it was a poor prognostic predictor of the patients [21]. In our study, further correlation analysis revealed that positive expression of YAP 1 was correlated closely with tumors poorer differentiation, higher pT and/or pN stages. Importantly, positive expression of YAP 1 was a strong and independent predictor of short overall survival of UCB patients, as evidenced by the Kaplan-Meier curves and multivariate Cox proportional hazards regression analysis. Furthermore, stratified survival analysis of UCB histopathological grade and/or pTN stage showed that YAP 1 expression was closely correlated to survival of certain subsets of UCB patients, including patients having grade 2/3 tumors and in pT1, pT2-4, pN- or pT2-4/ pN- stage. Thus, YAP 1 expression appears to have the potential to indicate certain outcomes in UCB patients. The examination of YAP 1 expression, therefore, could be used as an additional tool in identifying patients at risk of UCB progression, and it may also be useful in optimizing individual UCB therapy management. These findings underscore the potentially important role of YAP 1 in the underlying biological mechanism involved in the development and/or progression of UCB.

With respect to the function of the YAP 1 gene, as a candidate oncogene, YAP 1 has been shown to be a potent regulator of cell growth. Overexpression of YAP 1 in the liver of transgenic mice could expand the liver mass from 5% of bodyweight to 25% and eventually lead to tumor growth [17]. Moreover, YAP 1 overexpression stimulates proliferation and increases the saturation cell density in monolayer cultures of NIH-3T3 cells [16]. Furthermore, overexpression of YAP 1 in NSCLC cell lines resulted in a marked increase in the cell growth rate, and overcame cell contact inhibition [21]. It is confirmed that YAP 1 overexpression in MCF10A cells triggered epithelial–mesenchymal transition (EMT) [12], which is often associated with cancer cell invasion and metastasis. Although we observed a positive association between YAP 1 expression and Ki-67 expression (a marker for cell proliferation) in our UCB cohort, the precise mechanisms that is ultimately involved in the oncogenic processes of UCB remains to be investigated. Nevertheless, our findings suggest the potential important role of YAP 1 in the control of UCB cell proliferation, an activity that might be responsible, at least in part, for the development and/or progression UCB.

## Conclusions

In this study, we describe, for the first time, the mRNA and protein expression patterns of YAP 1 in human UCB tissues and in normal bladder tissues. Our results provide a basis for the concept that increased expression of YAP 1 in UCB may be important in the acquisition of an aggressive and/or poor prognostic phenotype. The results suggest that the expression of YAP 1, as examined by IHC, could be used as an important molecular marker for shortened survival time in patients with UCB, and it might be helpful to render a more tailored treatment strategy in this human cancer.

## Abbreviations

YAP 1: Yes-associated protein 1; UCB: Urothelial carcinoma of bladder; qRT-PCR: Quantitative real-time polymerase chain reaction; IHC: Immunohistochemistry; UC: Urothelial carcinoma; EMT: Epithelial–mesenchymal transition; RC: Radical cystectomy; TURBT: Transurethral resection of bladder tumor; TMA: Tissue microarray; H&E: Hematoxylin and eosin; EDTA: Ethylene-diamine tetraacetic acid; DAB: 3,3-diaminobenzidine; LI: Labeling index.

## Competing interests

The authors declare that they have no competing interests.

## Authors’ contributions

JYL evaluated the clinical records, carried out the experimental work and drafted the manuscript. YHL and HXL contributed for data interpretation and drafted the manuscript. YJL participated in the statistical analysis and help to draft the manuscript. SJM and JXZ help to carry out the immunohistochemistry assays. HFK contributed for critical revision of statistical analysis and of the manuscript. ZWL, ZLZ and LJJ critically revised the manuscript. FJZ designed the study and participated in its coordination. YXZ and DX participated in the design of the study, in its analysis and in the interpretation of the data. DX also participated in evaluated the immunohistochemistry results and wrote the manuscript. All authors read and approved the final manuscript.

## Pre-publication history

The pre-publication history for this paper can be accessed here:

http://www.biomedcentral.com/1471-2407/13/349/prepub
